# Altered Regional Brain Glucose Metabolism in Diffuse Large B-Cell Lymphoma Patients Treated With Cyclophosphamide, Epirubicin, Vincristine, and Prednisone: An Fluorodeoxyglucose Positron Emission Tomography Study of 205 Cases

**DOI:** 10.3389/fnins.2022.914556

**Published:** 2022-06-15

**Authors:** Yuxiao Hu, Qin Zhang, Can Cui, Yun Zhang

**Affiliations:** ^1^Department of PET/CT Center, Jiangsu Cancer Hospital, Jiangsu Institute of Cancer Research, The Affiliated Cancer Hospital of Nanjing Medical University, Nanjing, China; ^2^Department of Thoracic Surgery, Jiangsu Cancer Hospital, Jiangsu Institute of Cancer Research, The Affiliated Cancer Hospital of Nanjing Medical University, Nanjing, China

**Keywords:** ^18^F-FDG, PET, brain glucose metabolism, chemotherapy, diffuse large B cell lymphoma

## Abstract

**Background:**

A growing number of neuroimaging studies reported that chemotherapy might impair brain functions, leading to persistent cognitive alterations in a subset of cancer patients. The present study aimed to investigate the regional brain glucose metabolism differences between diffuse large B cell lymphoma (DLBCL) patients treated with cyclophosphamide, epirubicin, vincristine, and prednisone and controls using positron emission tomography with ^18^F-labeled fluoro-2-deoxyglucose integrated with computed tomography (^18^F-FDG PET/CT) scanning.

**Methods:**

We analyzed ^18^F-FDG PET data from 205 right-handed subjects (for avoiding the influence of handedness factors on brain function), including 105 post-chemotherapy DLBCL patients and 100 controls. The two groups had similar average age, gender ratio, and years of education. First, we compared the regional brain glucose metabolism using a voxel-based two-sample *t*-test. Second, we compared the interregional correlation. Finally, we investigated the correlations between the regional brain glucose metabolism and the number of chemotherapy cycles.

**Results:**

Compared with the controls, the post-chemotherapy group showed higher metabolism in the right hippocampus and parahippocampal gyrus (region of interest (ROI) 1) and the left hippocampus (ROI 2), and lower metabolism in the left medial orbitofrontal gyrus (ROI 3), the left medial superior frontal gyrus (ROI 4), and the left superior frontal gyrus (ROI 5). The two groups had different interregional correlations between ROI 3 and ROI 5. In some brain regions—mainly located in the bilateral frontal gyrus—the number of chemotherapy cycles was positively correlated with the regional brain glucose metabolism. Meanwhile, in some bilateral hippocampus regions, these two parameters were negatively correlated.

**Conclusion:**

The present study provides solid data on the regional brain glucose metabolism differences between post-chemotherapy DLBCL patients and controls. These results should improve our understanding of human brain functions alterations in post-chemotherapy DLBCL patients and suggest that ^18^F-FDG PET/CT scanning is a valuable neuroimaging technology for studying chemotherapy-induced brain function changes.

## Introduction

Diffuse large B-cell lymphoma (DLBCL) is the most common form of non-Hodgkin lymphoma worldwide. Nowadays, the immunochemotherapy regimen, rituximab combined with cyclophosphamide, doxorubicin, vincristine, and prednisone (R-CHOP), is the standard first-line DLBCL treatment. This treatment significantly improved clinical management, and allowed a high response rate with more than half of patients (Bouroumeau et al., 2021; Kimani et al., 2021; Wilson et al., 2021). With the prolonged survival of DLBCL patients, chemotherapy-related cognitive impairment (CRCI) has been attracting more attention in recent years. CRCI is commonly observed in chemotherapy-treated cancer patients. It is usually characterized by the impairment of memory, attention, learning, executive function, and processing speed (Lange et al., 2019; Du et al., 2021; Juan et al., 2022). So far, the neural mechanism of CRCI was not clear, but many researchers considered that it might related to the changes of brain structure and function in post-chemotherapy cancer patients (Wefel et al., 2015; Sousa et al., 2020).

Various multi-modality neuroimaging techniques showed objective alterations to explain these chemotherapy-induced brain disorders. Most of magnetic resonance imaging (MRI) studies involved breast cancer survivors. Structural MRI revealed reductions in gray matter volume, especially in the prefrontal cortex (Koppelmans et al., 2012; Conroy et al., 2013; McDonald and Saykin, 2013). Similarly, blood oxygen level-dependent functional MRI (BOLD fMRI) studies have observed alterations of functional connection patterns in the prefrontal cortex during the execution of working memory tasks and at the resting state (McDonald et al., 2012; Conroy et al., 2013; Chen et al., 2019).

^18^F-Fluorodeoxyglucose (FDG) Positron emission tomography (PET) is widely used on staging and response assessment in DLBCL. The PET studies of response assessment are conducted at various time points, including during and after the chemotherapy (Adams and Kwee, 2016; Frood et al., 2021). In addition to its application in the field of brain tumor (Boellaard et al., 2015; Treglia et al., 2019; Pietrzak et al., 2021), PET is a functional imaging modality with unique properties. It is conventionally used in brain science (Hu et al., 2015; Tan et al., 2022). PET can quantify some physiological parameters, such as cerebral blood flow, brain glucose metabolism, and brain receptor density. For example, using O-15 water PET scanning on adjuvant chemotherapy-treated breast cancer survivors performing a short-term recall task revealed significant cerebral blood flow alterations (Silverman et al., 2007). Additionally, FDG PET scanning can accurately depict regional brain glucose metabolism. Using FDG PET, Ponto et al. (2015) found that chemotherapy-treated breast cancer survivors had significantly lower FDG uptake in bilateral orbital frontal regions than control subjects.

However, most studies focused on the outcomes of brain structure and functions alterations on breast cancer survivors after chemotherapy rather than lymphoma survivors. Using brain resting state FDG PET, Baudino et al. (2012) observed that chemotherapy-treated lymphoma survivors had lower glucose metabolism than controls in bilateral prefrontal cortices, bilateral cerebellum, bilateral medial cortices, and bilateral limbic brain regions. A recent study revealed hypometabolic areas in the insular cortex, lateral frontal lobe, and posterior cingulate cortex in children with Hodgkin’s lymphoma after two chemotherapy cycles (Tauty et al., 2019). However, due to their limited sample sizes, these two studies might only capture relatively remarkable changes in regional brain glucose metabolism in chemotherapy-treated lymphoma patients. Thus, the present study re-addressed the differences in regional brain glucose metabolism between post-chemotherapy DLBCL patients and healthy controls using a large FDG-PET dataset (205 subjects).

## Materials and Methods

### Participants

One hundred and five DLBCL patients after chemotherapy (57 females/48 males, age: 50.48 ± 15.59 years, years of education: 8.99 ± 3.73 years) were enrolled at the PET center of Jiangsu Cancer Hospital, Jiangsu Institute of Cancer Research, The Affiliated Cancer Hospital of Nanjing Medical University between June 2018 and May 2021. The purpose of these FDG PET scans in the present study was not just to investigate the alteration of regional brain glucose metabolism in post-chemotherapy DLBCL survivors. They were performed for clinical restaging and therapeutic efficacy evaluation. The exclusive criteria for patients were as follows: (1) The time interval between the FDG PET scanning and the end of the last chemotherapy was more than 6 weeks. (2) Left-handed subjects. (3) Subjects with neurological or psychiatric disorders, alcohol or substance abuse history, serious cardiovascular and cerebrovascular diseases, poorly controlled diabetes, or digestive system diseases (excluded by the clinical examination and the case history). (4) Image with defect or artifacts in visual evaluation. Moreover, 100 controls (48 females/52 males, age: 51.96 ± 12.40 years, years of education: 10.03 ± 4.47 years) were enrolled at same center. These subjects underwent FDG PET scans for the purpose of a health examination. Exclusion criterion 2–4 was applied to the control group. Flow chart of subjects include showed in the Supplementary Material.

The two groups had similar average age, gender ratio, and years of education (Table 1). All subjects were ethnically Chinese. The 205 subjects underwent a whole-body FDG PET scan consisting of body scanning and brain scanning. This study only used brain scanning data. All participants provided written informed consent. The ethical committee of Nanjing Medical University approved this study.

**TABLE 1:** Demographic characteristics.

	DLBCL patients after chemotherapy (*n* = 105)	Controls (*n* = 100)	*p*-value
Gender (Female/male)	57/48	48/52	0.368 (χ^2^ = 0.81)
Age (years)	50.48 ± 15.59	51.96 ± 12.40	0.451 (*t* = –0.76)
Education (years)	8.99 ± 3.73	10.03 ± 4.47	0.073 (*t* = –1.81)

The time interval between the FDG PET scanning and the end of the last chemotherapy was 23.38 ± 9.37 for the DLBCL patients. All the DLBCL patients received the CHOP chemotherapy regimen (cyclophosphamide 750 mg/m^2^, epirubicin 60 mg/m^2^, and vincristine 1.4 mg/m^2^ intravenously on the first day and prednisone 100 mg/m^2^ orally on days 1–5). Total patients also received the monoclonal antibody rituximab (375 mg/m^2^) 1 day before chemotherapy. Among the 105 DLBCL patients, 11 (10.5%) received one chemotherapy cycle, 25 (23.8%) received two cycles, 12 (11.4%) received three cycles, 22 (21.0%) received four cycles, 10 (9.5%) received five cycles, and the remaining 25 (23.8%) received six cycles.

### Fluorodeoxyglucose Positron Emission Tomography Imaging Acquisition

PET/CT imaging was performed according to the guidelines (Boellaard et al., 2015). All participants fasted for at least 6 h before the FDG injection. We checked that blood glucose levels were between 3.9 and 6.3 mmol/L for subjects without diabetes and lower than 11.1 mmol/L for subjects with diabetes. Each subject received FDG intravenously (about 3.7–7.4 MBq/kg, equivalent to 0.1–0.2 mCi/kg). Then, all participants rested for 50–70 min in a dedicated, quiet, and dark waiting room for avoiding the visual and auditory stimulation. All of the subjects were required to remain in a supine position with closed eyes prior to the PET/CT scan. Each whole-body FDG PET scan for the subject included a body scan and a brain scan. All brain PET scans were performed after the body scan.

PET brain images were acquired using a PET/CT scanner (Discovery 710; General Electric Medical Systems, Milwaukee, WI, United States) with a non-contrast CT transmission scan (tube voltage, 120 kV; tube current, 300 mA, rotation time, 1S). The emission scan time was 5 min/bed position and one bed position covered the scanning range. The PET image data sets were reconstructed iteratively using the ordered subsets expectation maximization algorithm with CT-based attenuation correction. The parameters were as follows: Sharp IR algorithm with the VUE Point FX (fully three-dimensional iterative reconstruction), a 192 × 192 matrix, 24 subset/2 iteration.

### Data Preprocessing

The FDG PET data were preprocessed using the SPM8 software^1^ running on MATLAB2008.^2^ First, all PET images were spatially normalized into the standard Montreal Neurological Institute (MNI) space and resampled at a resolution of 2 × 2 × 2 mm^3^. Then, the normalized PET images were smoothed with an isotropic Gaussian kernel of 8 mm Full Width Half Maximum. Finally, the PET images were normalized by the mean signal of the entire brain from the original images.

### Data Analysis

#### Comparing Regional Brain Glucose Metabolism of Chemotherapy-Treated Diffuse Large B Cell Lymphoma Patients and Controls

To investigate the brain glucose metabolism alteration in post-chemotherapy DLBCL patients. Voxel-wise two samples *t*-test were carried out between post-chemotherapy DLBCL patients and controls using SPM8. We regarded differences as significant if the *p* < 0.05 at the voxel level and *p* < 0.05 FDR corrected for cluster level.

#### Interregional Relationships of Regional Brain Glucose Metabolism by Measuring Covariance Across Subjects

To assess whether chemotherapy co-modulated the regional brain glucose metabolism of different brain regions, we performed the following analysis. First, we selected regions of interest (ROIs) based on significant clusters of the post-chemotherapy group and controls group comparison results. This research method could also be seen in some previous literatures (Hu et al., 2013; Zhang et al., 2017). Next, we extracted the averaged regional brain glucose metabolisms within the five ROIs of all the participants. We then performed correlation analyses using the values of each pair of ROIs according to Schwarzkopf et al.’s (2012) method. This method detects outliers by first bootstrapping the Mahalanobis distance of each data point from the bivariate mean and then excluding all observations whose distance is ≥ 6. Finally, we investigated differences in interregional correlation by performing a linear-interaction analysis using the SurfStat Toolbox.^3^ The statistical threshold for ROI analyses was set at uncorrected *P* < 0.05.

#### Correlation With the Number of Chemotherapy Cycles

We investigated the voxel-wise correlations between regional brain glucose metabolism and the number of chemotherapy cycles. We set the following thresholds at uncorrected *p* < 0.01 for this exploratory analysis.

## Results

### Regional Brain Glucose Metabolism Comparison

**FIGURE 1:**
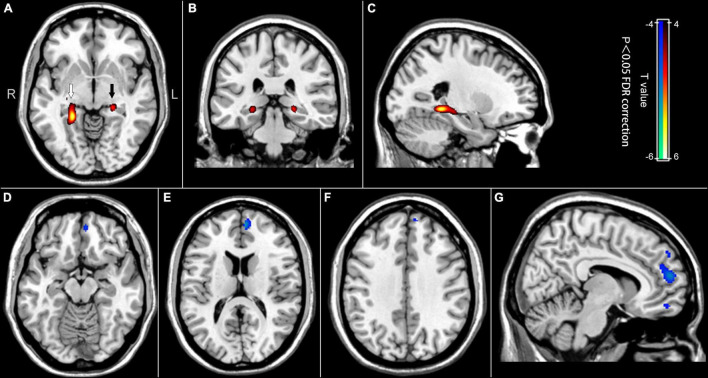
Difference maps of regional brain glucose metabolism between the post-chemotherapy group and the control group. Red to yellow areas represent the regions where the brain glucose metabolism values were higher in the post-chemotherapy group, while blue to cyan areas represent the opposite (*p* < 0.05, FDR correction). **(A)** Axial image showing the ROI 1 (white arrow) in the right hippocampus and parahippocampal gyrus, and ROI 2 (black arrow) in the left hippocampus. **(B)** Coronal image showing the ROI 1 and the ROI 2. **(C)** Sagittal image showing the ROI 1. **(D)** Axial image showing the ROI 3 in the left medial orbitofrontal gyrus. **(E)** Axial image showing the ROI 4 in the left medial superior frontal gyrus. **(F)** Axial image showing the ROI 5 in the left superior frontal gyrus. **(G)** Sagittal image showing ROI 3, 4, and 5 from bottom to top.

Figure 1 and Table 2 present the significant differences in regional brain glucose metabolism revealed by voxel-based analysis between two groups. Compared with the controls, the patients showed higher glucose metabolism in bilateral hippocampus [including the right hippocampus and parahippocampal gyrus (ROI 1) and the left hippocampus (ROI 2)], and lower glucose metabolism in medial frontal area [including the left medial orbitofrontal gyrus (ROI 3), left medial superior frontal gyrus (ROI 4), and left superior frontal gyrus (ROI 5)].

**TABLE 2:** The difference of regional brain glucose metabolism between chemotherapy-treated DLBCL patients and controls.

Brain regions	Voxels	P_*FDR*–*Corr*_	Peak	MNI coordinate		
			*t*-value	x	y	z
Patients > controls:						
ROI 1: Right hippocampus Right parahippocampal gyrus ROI 2: Center hippocampus	362 91	0.000 0.000 0.000	5.77 4.66 4.84	24 24 –22	–40 –28 –30	–6 –8 –6
Patients < controls:						
ROI 3: Left medial orbitofrontal gyrus ROI 4: Left medial superior frontal gyrus ROI 5: Left superior frontal gyrus	33 172 21	1.000 1.000 1.000 1.000 1.000	–4.00 –3.97 –3.97 –3.98 –3.99	–6 –12 –8 –6 –12	48 58 46 54 50	–18 14 28 6 38

### Interregional Correlation Comparison

**FIGURE 2:**
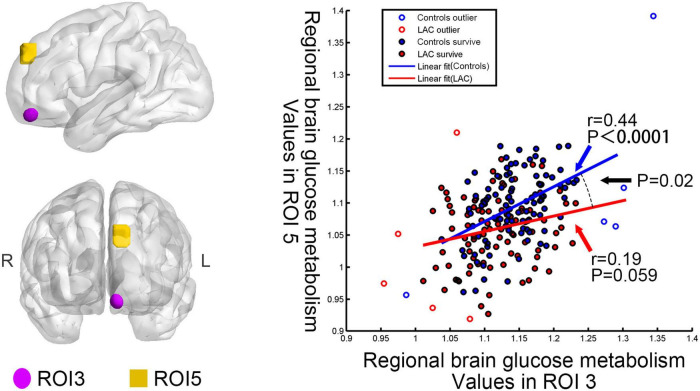
Differences in interregional correction between the post-chemotherapy group and the control group. Regional brain glucose metabolism values in the ROI 3 correlated with those in the ROI 5 for the control group (*r* = 0.44, *p* < 0.0001). No significant correlation emerged in the post-chemotherapy group (*r* = 0.19, *p* = 0.059). The interaction linear-model analysis showed that the post-chemotherapy group and the control group had different interregional correlation of regional brain glucose metabolism values (uncorrected *p* < 0.05). LAC: lymphoma patients after chemotherapy.

The patients and controls had different interregional correlation of brain glucose metabolism values between left medial orbitofrontal gyrus (ROI 3) and left superior frontal gyrus (ROI 5) (uncorrected *p* < 0.05). The interregional correlation analyses showed that the brain glucose metabolism values in the ROI 3 and ROI 5 were positively correlated in the health group (regression coefficient *r* = 0.44, *p* < 0.0001) but uncorrelated in the post-chemotherapy group (*r* = 0.19, *p* = 0.059) (Figure 2). Besides, we found no significant differences in the correlations on the other pairs of ROIs between the two groups.

### Correlation Analysis With the Number of Chemotherapy Cycles

**FIGURE 3:**
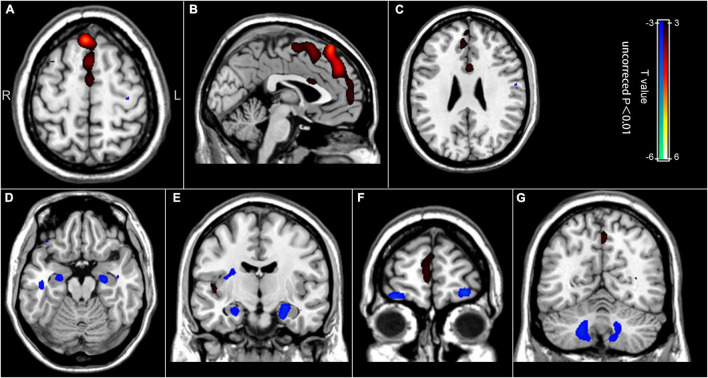
Correlation between the regional brain glucose metabolism values and the number of chemotherapy cycles. **(A–C)** Positive correlation between the number of chemotherapy cycles and the regional brain glucose metabolism in the bilateral medial superior frontal gyrus, the bilateral superior frontal gyrus, the right middle frontal gyrus, the bilateral anterior cingulate cortex, and the bilateral supplementary motor area. **(D–G)** Negative correlation between the number of chemotherapy cycles and the regional brain glucose metabolism in the bilateral hippocampus, the bilateral orbitofrontal cortex, and the bilateral cerebellum.

In the chemotherapy group, we observed significant positive correlations between the regional brain glucose metabolism and the number of chemotherapy cycles in the bilateral medial superior frontal gyrus, the bilateral superior frontal gyrus, the right middle frontal gyrus, the bilateral anterior cingulate cortex, and the bilateral supplementary motor area. Besides, we noted negative correlations in the bilateral hippocampus, the bilateral orbitofrontal cortex, and the bilateral cerebellum (uncorrected *p* < 0.01) (Table 3 and Figure 3). These brain regions largely overlapped with those in the group comparison, demonstrating consistency in the brain regions involved.

**TABLE 3:** Correlations between the regional brain glucose metabolism and the number of chemotherapy cycles in the post-chemotherapy group.

Positive correlation:	Negative correlation:
Bilateral medial superior frontal gyrus; Bilateral superior frontal gyrus; Right middle frontal gyrus; Bilateral anterior cingulate cortex; Bilateral supplementary motor area.	Bilateral hippocampus; Bilateral orbitofrontal cortex; Bilateral cerebellum.

## Discussion

Based on the large FDG-PET data set from 105 DLBCL patients treated with chemotherapy and 100 controls, we investigated the chemotherapy-induced regional brain glucose metabolism alterations in DLBCL survivors. Our main findings are: (I) The post-chemotherapy DLBCL patients showed higher FDG uptake than controls, mainly in the bilateral hippocampal regions, and lower FDG uptake in the left medial frontal gyrus. (II) The post-chemotherapy and control groups had significantly different interregional glucose metabolic correlations between ROI 3 and ROI 5. (III) Some brain regions showed positive correlations with the number of chemotherapy cycles, mainly in the bilateral frontal gyrus. Meanwhile, the bilateral hippocampus, the bilateral orbitofrontal cortex, and the bilateral cerebellum displayed negative correlations with the number of chemotherapy cycles.

Both animal and preclinical clinical studies have revealed that the hippocampus and the frontal lobes are particularly vulnerable to the adverse effects of chemotherapy (Winocur et al., 2018; Peukert et al., 2020). As early as 2004, Rzeski et al. (2004) found that cytotoxic drugs could extensively damage the hippocampal dentate gyrus of 7-day-old rats. Then, Acharya et al. (2015) observed that treating mice with cyclophosphamide significantly decreased granule and pyramidal cell counts in the dentate gyrus and CA1 regions of the hippocampus. Using multi-modality neuroimaging techniques, some preclinical studies have found that chemotherapy significantly changed brain volume and regional brain glucose metabolism, mainly in the hippocampus and frontal lobes (Winocur et al., 2018; Lee et al., 2019).

Similarly, clinical studies have revealed significant differences in gray and white matter volume, white matter connections, brain regions activation, cerebral blood flow, and regional brain glucose metabolism within the hippocampus, parahippocampus, and frontal lobes in post-chemotherapy breast cancer survivors (Silverman et al., 2007; Koppelmans et al., 2012; McDonald et al., 2012; Conroy et al., 2013; McDonald and Saykin, 2013; Ponto et al., 2015; Chen et al., 2019). Besides, some ^18^F-FDG PET studies focused on post-chemotherapy Hodgkin’s lymphoma survivors and found significant abnormalities in whole-brain and regional-brain glucose metabolism (Baudino et al., 2012; Sorokin et al., 2014; Chiaravalloti et al., 2015; Tauty et al., 2019).

In line with previous studies, the present study showed decreased regional brain glucose metabolism in the left medial frontal gyrus of chemotherapy-treated DLBCL survivors. Using a large FDG-PET data set, we confirmed the results of the previous studies and enhanced their reliability. In general, the blood-brain barrier prevents chemotherapeutic agents from reaching the brain, but animal studies revealed that some chemotherapeutic agents (for example, cyclophosphamide and doxorubicin) were associated with decreased neurogenesis (Rzeski et al., 2004). Moreover, some researchers observed changes in the structure and function of the medial frontal gyrus in patients with different psychiatric conditions. For example, Niida et al. (2012) revealed gray matter volume reduction in the bilateral orbitofrontal cortex of patients with major depressive disorders. In addition, post-traumatic stress disorder patients have a significantly reduced cerebral blood flow in the frontal lobe (Liu et al., 2016). Therefore, the regional brain glucose metabolism reduction that we observed in the left medial frontal gyrus might be due to the toxicity of the chemotherapeutic drugs and the acute anxiety status of the DLBCL patients (Chiaravalloti et al., 2015).

Chemotherapy can produce diverse hippocampal structure and function changes. Some studies reported overall volume reduction, functional connections decrease, and deformation in the hippocampus of chemotherapy-treated patients (Peukert et al., 2020). Meanwhile, others found increased hippocampus activation during verbal memory tests (Lopez Zunini et al., 2013). The present study showed that chemotherapy-treated DLBCL patients had higher FDG uptake in the bilateral hippocampal regions than the controls. This inconsistency might be due to the different types of cancers, the different types of chemotherapeutic drugs used, and different cancer course progression at the time of scanning.

It is worth noting that our research found a covariance of regional brain glucose metabolism values between ROI 3 (the left medial orbitofrontal gyrus) and ROI 5 (the left superior frontal gyrus) in the controls. This result might imply the coordination of these two brain regions in healthy people. However, we did not observe significant correlations in the post-chemotherapy group. The post-chemotherapy group and the control group had significantly different interregional correlations between these two ROIs. This finding might indicate that the chemotherapeutic drugs spatially affected the pattern of brain glucose metabolism (Zhang et al., 2017).

The present study also showed that the chemotherapy cycles were positively correlated with the regional brain glucose metabolism of the bilateral medial superior frontal gyrus and bilateral superior frontal gyrus, and negatively correlated with that in the bilateral hippocampus. Remarkably, the post-chemotherapy group had lower regional brain glucose metabolism in the ROI 4 (the left medial superior frontal gyrus) and ROI 5 (the left superior frontal gyrus) and higher glucose uptake in the ROI 1 (the right hippocampus and parahippocampal gyrus) and ROI 2 (the left hippocampus). These observations suggest that the regional brain glucose metabolism alterations are more marked after the first cycle of the chemotherapy in these regions (the left medial superior frontal gyrus, the left superior frontal gyrus, and the bilateral hippocampus). Besides, increasing the number of cycles seemed to partially restore brain glucose metabolism. This observation is consistent with previous studies (Baudino et al., 2012; Chiaravalloti et al., 2015; Wagner et al., 2020).

The BOLD fMRI data and FDG PET data reflect the different physiological information. BOLD fMRI relies on a surrogate signal, resulting from oxygen metabolism, cerebral blood flow, cerebral blood volume, and regional tissue characteristics. FDG PET directly reflects the brain glucose metabolism. However, both of them do not directly measure neural activity. It was widely known that BOLD fMRI was most commonly used in the field of brain science and could be applied in evaluating the alterations of brain function in CRCI patients (Conroy et al., 2013; Chen et al., 2019). Our study draws a panorama of chemotherapy-related brain metabolic regional alteration, suggesting that FDG PET is also a suitable tool for evaluating chemotherapy brain.

There are several limitations in the present study. First, we used cross-sectional data from the post-chemotherapy DLBCL patients and did not obtain data before the chemotherapy. By comparison, a longitudinal study directly comparing the regional brain glucose metabolism of a control group, a pre-chemotherapy group, and a post-chemotherapy group would be more accurate. Second, we did not consider the co-occurrence of DLBCL and some psychiatric disorders. In fact, cancer diagnosis and treatment are often accompanied by many acute and chronic stressors, which can affect regional brain glucose metabolism in some brain regions. To address this problem, a few psychiatrists will join our team in a follow-up study. Finally, the regional brain glucose metabolism alterations observed in the chemotherapy-treated DLBCL patients might be due to specific cognitive and behavioral abnormalities. The lack of neuropsychological and psychiatric evaluation before and after chemotherapy is, therefore, an obvious limitation. Future ^18^F-FDG PET studies should integrate neuropsychological or psychiatric evaluations before and after the chemotherapy.

In summary, the present study has produced a large ^18^F-FDG PET data set supporting the hypothesis that cyclophosphamide, epirubicin, vincristine, and prednisone would change regional brain glucose metabolism in some brain regions of DLBCL patients, mostly in the hippocampus, and frontal lobes. The abnormal brain regions revealed in this study partially overlapped with the abnormal brain regions of CRCI reported in the previous literatures (Kiesl et al., 2022). Therefore, we speculated that the DLBCL survivors treated with CHOP might have CRCI. This hypothesis needs further research. To this day, the management of CRCI remains a clinical challenge. Some previous studies suggested that probiotics supplement and a long-term supervised exercise intervention program might prevent the occurrence of CRCI (Juan et al., 2022; Kiesl et al., 2022). Besides, our study showed that ^18^F-FDG PET/CT scanning was a valuable tool to study functional alteration in the brain, providing objective and accurate quantitative information on the severity and location of the affected sites.

## Data Availability Statement

The original contributions presented in this study are included in the article/Supplementary Material, further inquiries can be directed to the corresponding author/s.

## Ethics Statement

The studies involving human participants were reviewed and approved by the Ethical Committee of Nanjing Medical University. The patients/participants provided their written informed consent to participate in this study.

## Author Contributions

YH and QZ: study concept and design, analysis, and interpretation of data. YH and CC: data collection. YH, QZ, and YZ: drafting and critical revision of the manuscript. All authors contributed to the article and approved the submitted version.

## Conflict of Interest

The authors declare that the research was conducted in the absence of any commercial or financial relationships that could be construed as a potential conflict of interest.

## Publisher’s Note

All claims expressed in this article are solely those of the authors and do not necessarily represent those of their affiliated organizations, or those of the publisher, the editors and the reviewers. Any product that may be evaluated in this article, or claim that may be made by its manufacturer, is not guaranteed or endorsed by the publisher.

## Footnotes

1
http://www.fil.ion.ucl.ac.uk/spm/

2
www.mathworks.com

3
https://www.math.mcgill.ca/keith/surfstat/
