# Supplementary figures and images for: Altered Regional Brain Glucose Metabolism in Diffuse Large B-Cell Lymphoma Patients Treated With Cyclophosphamide, Epirubicin, Vincristine, and Prednisone: An Fluorodeoxyglucose Positron Emission Tomography Study of 205 Cases
Source: Front Neurosci. 2022 Jun 15;16:914556. doi: 10.3389/fnins.2022.914556 (PMC9240384; doi:10.3389/fnins.2022.914556)

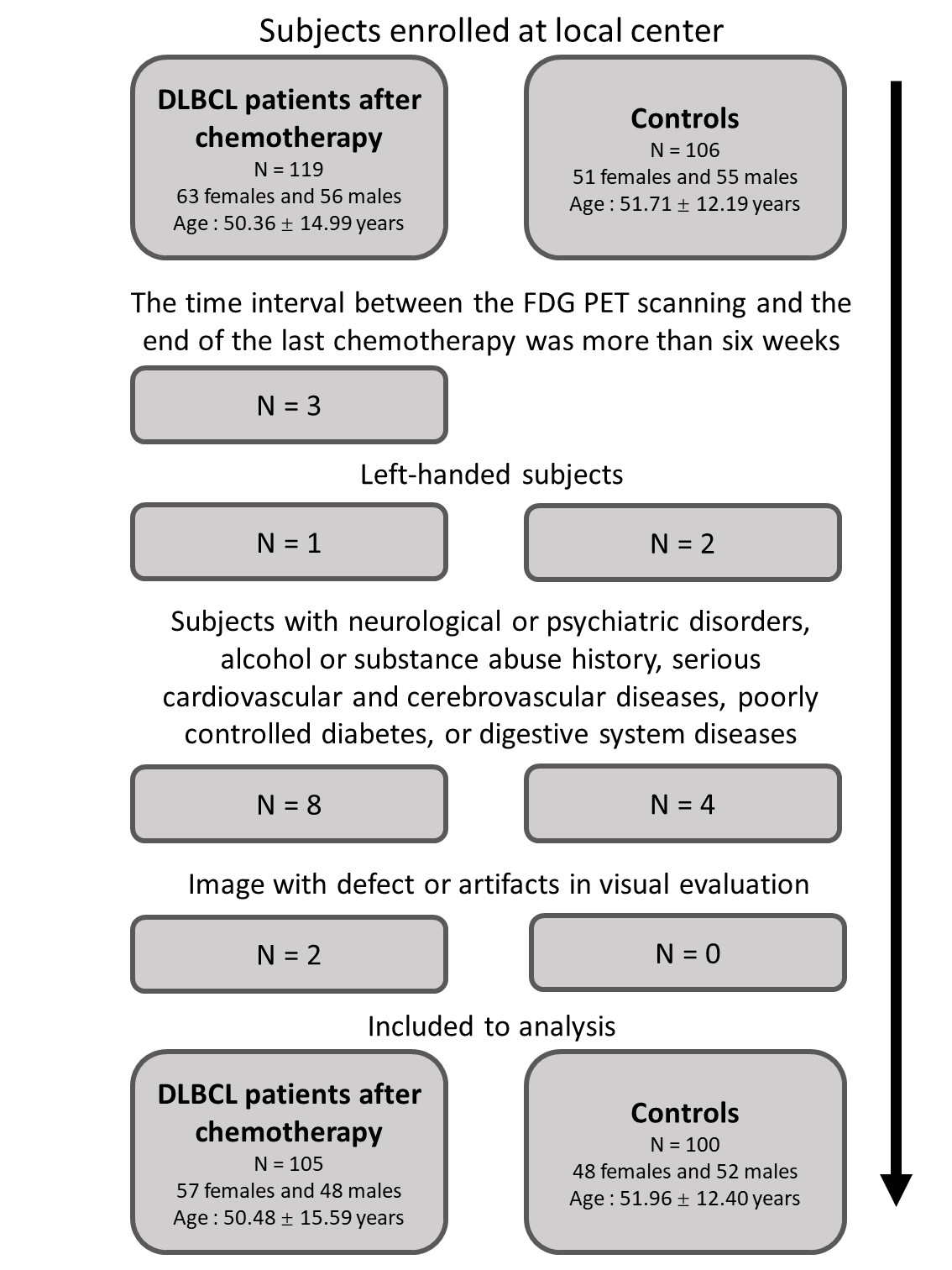

Supplement: Supplementary file 1 [file Data_Sheet_1.docx]
